# Z944, a Novel Selective T-Type Calcium Channel Antagonist Delays the Progression of Seizures in the Amygdala Kindling Model

**DOI:** 10.1371/journal.pone.0130012

**Published:** 2015-08-14

**Authors:** Pablo Miguel Casillas-Espinosa, Ashleigh Hicks, Amy Jeffreys, Terrance P. Snutch, Terence J. O’Brien, Kim L. Powell

**Affiliations:** 1 The Department of Medicine, The University of Melbourne, Royal Melbourne Hospital, Melbourne, Australia; 2 Michael Smith Laboratories, University of British Columbia, Vancouver, BC, Canada; University of Waterloo, CANADA

## Abstract

Temporal lobe epilepsy (TLE) is the most common form of drug resistant epilepsy. Current treatment is symptomatic, suppressing seizures, but has no disease modifying effect on epileptogenesis. We examined the effects of Z944, a potent T-type calcium channel antagonist, as an anti-seizure agent and against the progression of kindling in the amygdala kindling model of TLE. The anti-seizure efficacy of Z944 (5mg/kg, 10mg/kg, 30mg/kg and 100mg/kg) was assessed in fully kindled rats (5 class V seizures) as compared to vehicle, ethosuximide (ETX, 100mg/kg) and carbamazepine (30mg/kg). Each animal received the seven treatments in a randomised manner. Seizure class and duration elicited by six post-drug stimulations was determined. To investigate for effects in delaying the progression of kindling, naive animals received Z944 (30mg/kg), ETX (100mg/kg) or vehicle 30-minutes prior to each kindling stimulation up to a maximum of 30 stimulations, with seizure class and duration recorded after each stimulation. At the completion of drug treatment, Ca_V_3.1, Ca_V_3.2 and Ca_V_3.3 mRNA expression levels were assessed in the hippocampus and amygdala using qPCR. Z944 was not effective at suppressing seizures in fully kindled rats compared to vehicle. Animals receiving Z944 required significantly more stimulations to evoke a class III (p<0.05), IV (p<0.01) or V (p<0.0001) seizure, and to reach a fully kindled state (p<0.01), than animals receiving vehicle. There was no significant difference in the mRNA expression of the T-type Ca^2+^ channels in the hippocampus or amygdala. Our results show that selectively targeting T-type Ca^2+^ channels with Z944 inhibits the progression of amygdala kindling. This could be a potential for a new therapeutic intervention to mitigate the development and progression of epilepsy.

## Introduction

Temporal lobe epilepsy (TLE) is the most common epilepsy syndrome that is resistant to drug treatment [1]. Most cases of TLE are believed to be acquired resulting from an insult to the brain, such as febrile seizures, head trauma, meningitis or stroke [2, 3]. TLE is characterized by complex partial seizures, which may progress to secondary generalization with tonic-clonic components [2].

The kindling model has been used extensively as a functional model of TLE. In kindling, repetitive stimulation results in a progressive increase in the severity and duration of the seizures [4, 5]. Once the rat has been kindled, the intensified response to the electrical stimulus seems to be permanent, indicating the development of chronic brain alterations [6]. However, the altered neuronal response develops in the absence of gross morphological damage [5]. In addition, kindling has been used for the preclinical evaluation of antiepileptic drugs. Anti-epileptic drugs which are clinically effective for TLE, such as benzodiazepines, phenytoin, valproate and carbamazepine (CBZ), have been demonstrated to inhibit kindled seizures [7, 8].

While the molecular mechanisms that occur in amygdala kindling resulting in increased seizure severity are not completely understood, several studies have associated changes in intracellular calcium (Ca^2+^) levels with the development of seizures [9–12]. In fact, an imbalance of Ca^2+^ homeostasis can change intrinsic electrical response of hippocampal neurons to a burst of several spikes, which may be a potential critical mechanism for the development of seizures [13–15].

The voltage-gated Ca^2+^ channels are transmembrane ion channels that allow the influx of Ca^2+^ into neurons and other cell types. Both high voltage-activated and low voltage-activated (T-type) Ca^2+^ channel subtypes mediate critical roles in regulating the function of neurons, including neurotransmitter release, gene transcription and membrane depolarization and excitability [16, 17]. The family of T-type Ca^2+^ channels comprises distinct genes encoding the Ca_V_3.1, Ca_V_3.2 and Ca_V_3.3 subunits. T-type Ca^2+^ channels exhibit a higher level of expression at cell bodies and dendrites where they contribute to the regulation of neuronal excitability [18]. T-type Ca^2+^ channels can affect paroxysmal burst-firing due to disruptions in Ca^2+^ homeostasis [19]. Moreover, Ca^2+^ currents have shown to be increased in rats after electrical kindling [20]. Pilocarpine-induced SE, another model of acquired TLE, resulted in an up-regulation of intrinsic bursting in hippocampal CA1 pyramidal cells driven by a Ca^2+^-dependent ionic mechanism after SE. This may be responsible for the initiation of epileptiform events that synchronize CA1 pyramidal cell activity [14]. Moreover in the same animal model, a selective and transient increase in Ca_V_3.2 mRNA expression in hippocampal CA1 pyramidal neurons [21] was found coupled with an up-regulation of T-type Ca^2+^ currents after SE [14, 15, 21, 22].

We have previously shown that Z944, a high-affinity pan-T-type Ca^2+^ channel antagonist, significantly inhibits absence seizures by 85 to 90% in the GAERS model of genetic generalized epilepsy [23]. Here we investigated the efficacy of Z944 in delaying the progression of amygdala kindling and as an anti-seizure agent in the amygdala kindling model of TLE.

## Methods

### Ethics Statement

All procedures on rats were approved by The University of Melbourne Animal Ethics committee (ethics numbers 1011823 and 0911543) and followed the Australian Code of Practice for the care and use of animals for scientific purposes.

### Surgery

Wistar rats aged 9–10 weeks were anaesthetized with 5% isoflurane. Three electroencephalogram (EEG) recording electrodes (open 20–24 AWG) soldered to screws (1.4x3 mm) were implanted just in contact with the dura mater. Each animal was implanted stereotaxically with a stimulating bipolar electrode in the left basolateral amygdaloid nucleus using the coordinates AP±3.0 mm, ML±5.9 mm, DV±6.0 mm [24]. Four hours after surgery and the following day, animals were given an intraperitoneal (IP) injection of 0.9% saline solution and a 0.1 ml/100 g IP dose of carprofen.

### Amygdala kindling procedure

Seven to 10 days post-surgery, the after discharge threshold (ADT) for each animal was determined by administering a stimulation (frequency = 60.1 Hz; duration = 1000 μs) at one minute intervals until a seizure was elicited. The amplitude of the stimulation was started at 0.02 mA with increments of 0.02 mA; the stimulations were stopped when the animal experienced a seizure of ≥6 second duration, or if the amplitude of 0.40 mA was reached without the occurrence of a seizure. The amplitude at which an animal exhibited a seizure was recorded and used for kindling stimulations throughout the experiment. If the animal did not exhibit a seizure by 0.40 mA, the stimulation was repeated 24 hours later up to a maximum of two repeats. Kindling was performed twice a day for five days per week with an endpoint of 30 stimulations or until the animal presented five class V seizures (referred to as fully kindled). The seizure severity was classified from 0 (less severe) to V (most severe) according to the Racine Scale [25]. Sham kindled animals were handled the same way as the kindled animals, although no electrical stimulation was applied. The stimulation protocol used in the present study does not lead to the development of spontaneous seizures. An animal is classified as fully kindled once it has experienced 5 class V seizures.

### Drug preparation

Z944 was dissolved in 10% DMSO and 90% Na^+^ carboxy-methylcellulose, ethosuximide (ETX) was dissolved in saline and CBZ was dissolved in 10% DMSO, 40% propylene glycol and 50% saline.

### Testing Z944 as an anti-seizure agent

Fully kindled rats (i.e. had experienced at least 5 Class V seizures and had a stable seizure response evoked by electrical stimulation) were used for this study (n = 7). Drug testing was performed twice a week until all animals had received each of the seven treatments, Z944 (5 mg/kg, 10 mg/kg, 30 mg/kg and 100 mg/kg), vehicle (10% DMSO/90% Na^+^-CMC or CBZ vehicle 20% DMSO/40% propylene glycol/ 40% saline), ETX (100 mg/kg) or CBZ (30 mg/kg). Prior to drug testing each day, animals were subjected to a maximum of three pre-drug stimulations 30 minutes apart. The animals qualified for testing on that particular day if a class IV or V seizure was elicited in at least 2 out of 3 pre-drug stimulations. Stimulations consisted of a 1 second train of 1ms biphasic square wave pulses at a frequency of 60 Hz with current amplitude based on each animal ADT that elicited a seizure. Thirty minutes after the last pre-drug stimulation, the animals received an IP injection of one of the seven treatments in a randomized manner at a dose volume of 5 ml/kg. Six post-drug stimulations were administered to each animal, the first stimulation was given 15 minutes post drug injection (to allow brain penetration of the drugs) and the following five stimulations were delivered in 30 minutes intervals thereafter with the seizure class elicited being recorded after each post-drug stimulation. The duration of the primary and total seizures, if any, were recorded. An investigator blinded to the drug treatment analyzed all EEG recordings. Primary after discharge is the duration of the seizure elicited from the kindling stimulation. Total after discharge is the total seizure duration including any secondary seizures that occur after the primary after discharge. Primary after discharge duration and total seizure duration were recorded and averaged for the 6 post-drug stimulations.

Adverse effects of each treatment were recorded, based on a scale of 0–4; where a score of 0 indicates no sedation, normal movement, a score of 1 is for slight sedation, slow movement but alert when startled, a score of 2 is for mildly sedated, struggles when restrained, a score of 3 shows a sedated animal that is not moving in cage, but does respond to provocation and, the highest score of 4 indicates an animal that is very sedate, catatonic and unable to stand when provoked [23], every 15 minutes from the first post-drug stimulation until 90 minutes and then every half hour thereafter until 150 minutes after the first post-drug stimulation. Animal weight and behaviour when handled were monitored for the duration of the experimental period. Additionally, animals were assessed by observation twice per day for any adverse effects of drugs on grooming, fur appearance, gait, and excretion.

### Testing Z944 in the progression of seizures in the amygdala kindling model

Wistar rats underwent surgery to implant bipolar stimulation electrodes and EEG recording electrodes as described above. ADT was determined as described for the anti-seizure study. Animals were randomly assigned to four different cohorts; Cohort 1: kindled sham + vehicle (n = 8); Cohort 2: kindled + vehicle (n = 6); Cohort 3: kindled + ETX (100 mg/kg, n = 6) and Cohort 4: kindled + Z944 (30 mg/kg, n = 7). Thirty minutes prior each kindling stimulation, the animals received an IP injection of vehicle, ETX, or Z944 administered at a dose volume of 5 ml/kg up to an endpoint of 30 stimulations and the seizure class elicited and seizure duration was recorded. None of the animals from the kindled sham + vehicle groups displayed any seizures and were therefore not included in seizure analysis. Adverse effects were recorded every 15 minutes from the drug administration until 90 minutes after. An investigator blinded to the drug treatment analyzed all EEG recordings. The seizure class each stimulation elicited was recorded and averaged and the duration of the primary and total seizures were recorded.

### Brain dissection

Two to four weeks after the completion of kindling, rats were anaesthetized with 5% isoflurane and a dose of 15 mg/100g body weight of pentobarbital Na^+^ was administered followed by the rapid extraction of the brain, and hippocampus and amygdala were dissected, snap frozen over liquid nitrogen and stored at -80°C.

### Quantitative PCR

RNA extraction was performed using the RNeasy Mini Kit (QIAGEN) following the manufacturer’s protocol and treated with DNase I (150 U, QIAGEN) to remove any contaminating DNA. 1 μg RNA was reverse transcribed to cDNA using the Omniscript RT Kit (QIAGEN) following the manufacturer’s protocol. Quantitative polymerase chain reaction (qPCR) was performed on 50 ng cDNA using catalogued Taqman gene expression assays for Ca_V_3.1 (Assay ID Rn00581051_m1, Applied Biosystems), total Ca_V_3.2 (Assay ID Rn01460348_m1, Applied Biosystems), Ca_V_3.3 (Assay ID Rn01505208_m1, Applied Biosystems) and custom designed Taqman gene expression assays for the Ca_V_3.2 splice variants: +exon 25 (H- Ca_V_3.2-plus25 forward primer, GCGCAGGAGCACTTTCC; H-Ca_V_3.2-plus25 reverse primer, AGTGTGTGAATAGTCTGCGTAGTA; H-Ca_V_3.2-plus25-Probe, CCAACCCAGAGGCCCAG);—exon 25 (H- Ca_V_3.2-minus25 forward primer, CGCCGGGAGGAGAAACG; H- Ca_V_3.2-minus25 reverse primer, AGTGTGTGAATAGTCTGCGTAGTA; H-Ca_V_3.2-minus25-Probe, CTGGGCCTTCCTGCGCC) [26]. Relative expression of the Ca_V_3.1, total Ca_V_3.2, Ca_V_3.3, Ca_V_3.2 splice variant +25 and -25 SV were compared to the geometric average of the mRNA levels of the housekeeping genes GAPDH (Assay ID: Rn01775763_g1) and SDHA (Assay ID: Rn00590475_m1). Analysis was performed using the ΔΔC_T_ method [27]. The average of the relative expression levels for each kindled group was compared with the control group (sham kindled + vehicle).

### Statistical Analysis

All statistical analyses were done using GraphPad Prism version 5. Kruskal-Wallis test with Dunns post-hoc test was used to analyze anti-seizure data (seizure class, seizure duration and adverse effects data). Two way ANOVA with Bonferroni correction post-hoc was used to analyze seizure class and seizure duration in the progression to kindling data. One-way ANOVA with Bonferroni correction post-hoc was used to analyze the number of seizures to reach fully kindled state and qPCR data.

## Results

### Z944 did not suppress seizures class in fully kindled animals

**Fig 1:**
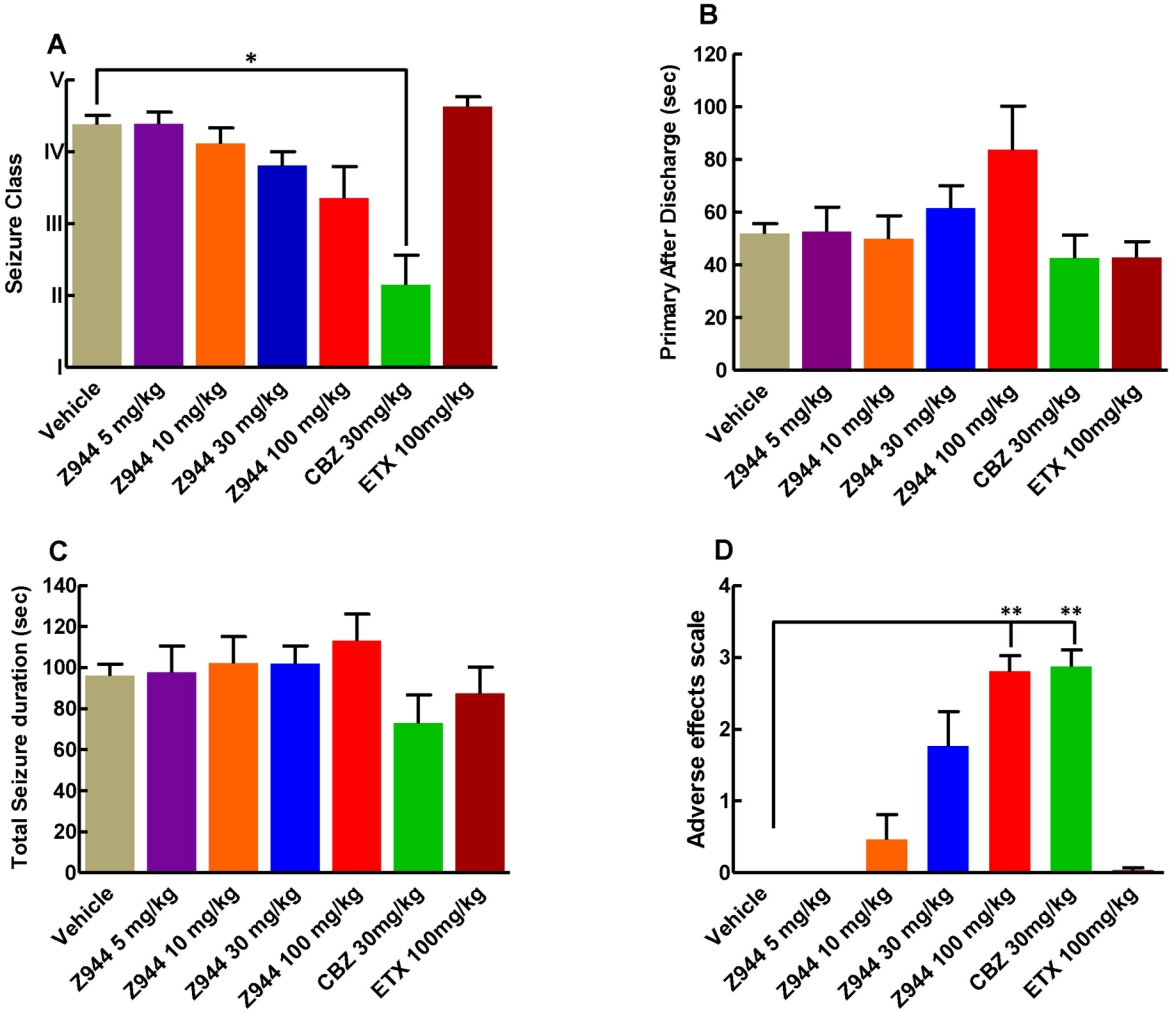
Z944 has no anti-seizure effect in fully kindled animals. (A) Z944 does not suppress seizures in fully kindled animals compared to vehicle treatment. Carbamazepine (CBZ, n = 7) significantly reduced the class of seizure elicited when compared to vehicle (p<0.05). (B) All four doses of Z944, ETX and CBZ did not affect the primary after discharge when compared to vehicle (p>0.5). (C) Total seizure duration was unaffected in all treatment groups. (D) Adverse effects of Z944 are dose dependent. Z944 (100 mg/kg) and CBZ showed significantly higher adverse effects when compared to vehicle, (p<0.01 for both treatments). *p<0.05, **p<0.01. Kruskal-Wallis test with Dunns post-hoc test.

There was no significant difference in seizure characteristics between CBZ vehicle and Z944 vehicle therefore the results from both vehicles were averaged to obtain a single vehicle value to be used as comparison for the different drug treatments. All four doses of Z944 did not significantly alter the seizure class elicited by kindling stimulations compared to vehicle in fully kindled rats (Fig 1A). As expected, CBZ, a drug used clinically to suppress partial seizures, significantly reduced seizure class when compared to vehicle (n = 7, p<0.05), while ETX, a drug that is ineffective clinically against partial seizures, had no effect on seizure class in fully kindled rats compared to vehicle (Fig 1A).

### Z944 has no effect on primary and total seizure duration

Animals treated with Z944 (n = 7, all four doses), CBZ (n = 7,) and ETX (n = 7) had no significant effect on the duration of the primary after discharge (the seizure elicited from the kindling stimulation) (Fig 1B) or total after discharge (i.e. total seizure duration) compared to vehicle treated animals (Fig 1C).

### Putative adverse effects of Z944 are dose-dependent

Animals treated with Z944 showed dose-dependent signs of adverse effects being statistically significant at the dose of 100 mg/kg (p<0.01) when compared to vehicle (n = 7,). Z944 at 100 mg/kg yielded an average adverse effect score of 2.8±0.59 which equates to the animals being sedate and with reduced movement around the cage. CBZ treatment resulted in significant adverse effects (p<0.01) compared to vehicle, which was similar to the 100 mg/kg dose of Z944. Animals showed no signs adverse effects following treatment with vehicle, ETX and Z944 (5 and 10 mg/kg). Animals treated with Z944 at a dose of 30 mg/kg showed milder signs, including slight sedation and slow movement (Fig 1D) but this was not statistically different from vehicle.

### Z944 treatment delays seizure progression in amygdala kindled animals

**Fig 2:**
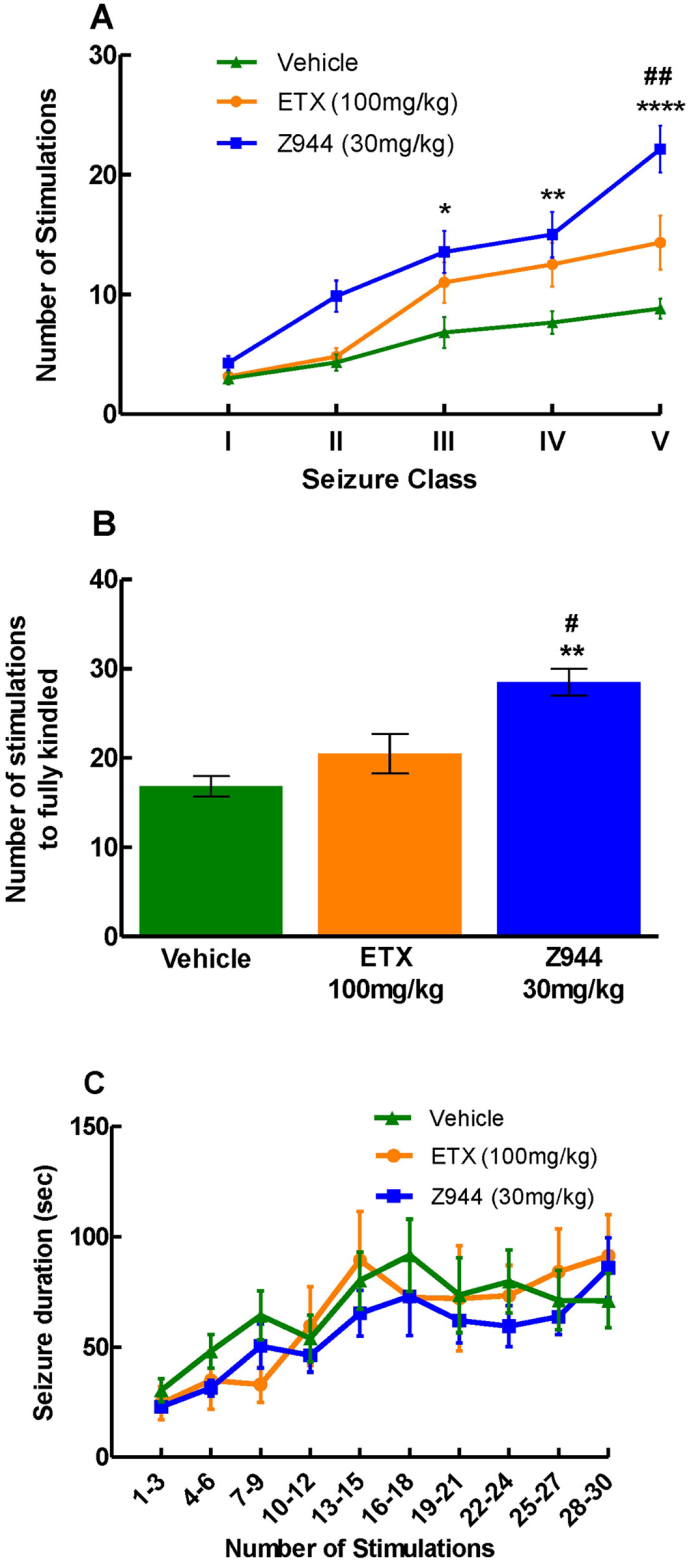
Z944 delays the progression to kindling. (A) Animals receiving Z944 (n = 7, 30 mg/kg) required significantly more stimulations to evoke a class III (p<0.05), IV (p<0.01) or V (p<0.0001) seizure than the animals receiving vehicle (n = 6) and required more stimulations to reach class V (p<0.01) when compared to ethosuximide (ETX, n = 6, 100 mg/kg) treated animals. (B) Z944 treated animals (n = 7, 30 mg/kg) required significantly more stimulations to reach the fully kindled state when compared to vehicle (n = 6, p<0.01) and ETX (n = 6, 100 mg/kg, p<0.05) treatment groups. (C) There was no significant difference in average seizure duration between the three treatment groups. *p<0.05, **p<0.01, ****p<0.0001 Z944 vs vehicle. #p<0.05, ##p<0.01 Z944 vs ETX. (A) and (C) Two way repeated measures ANOVA with Bonferroni post-hoc test. (B) One way ANOVA with Bonferroni post-hoc test.

Z944 (n = 7, 30 mg/kg) significantly delayed the progression of kindling when compared to ETX (n = 6) and vehicle (n = 6) (Fig 2A). Z944 treated animals required a significantly greater number of stimulations to reach class III (p<0.05), class IV (p<0.01) and class V (p<0.0001) seizures when compared to vehicle treated animals. Moreover, in comparison with ETX treated animals, Z944 treated animals required more stimulations to evoke class V seizures (p<0.01). Animals receiving Z944 showed mild adverse effects (score of 1, slight sedation, slow movement, but alert when startled). These mild effects were only seen during the first week of kindling (data not shown).

The number of stimulations required to reach a fully kindled state was also evaluated (Fig 2B). Vehicle treated animals required 17±3 stimulations, whereas animals receiving ETX needed 21±5 stimulations to reach the fully kindled state. Z944 treated animals showed a slower progression to the fully kindled state. Interestingly, only one out of seven Z944 treated animals reached the fully kindled state within 30 stimulations, whereas all of the ETX (n = 6) and vehicle treated (n = 6) animals became fully kindled. Animals who received 30 stimulations without reaching the fully kindled state were assigned the maximum value of 30. One way ANOVA with Bonferroni post hoc test showed that Z944 treated animals required significantly more stimulations to reach a fully kindled state when compared to vehicle (p<0.01) and ETX (p<0.05,Fig 2B).

### Z944 treatment does not affect seizure duration

The duration of the primary discharge (the seizure elicited from the kindling stimulation), was also evaluated for the progression to kindling experiments. Two-way repeated measures ANOVA with a Bonferroni multiple comparisons post hoc test showed that there was no significant difference in the duration of the primary discharge between the three treatment groups (Fig 2C).

### Z944 treatment does not affect T-type calcium channel mRNA expression in the amygdala or hippocampus

The 3 T-type calcium channels (Ca_v_3.1, Ca_v_3.2 and Ca_v_3.3) and the two of the major Ca_V_3.2 splice variants (Ca_V_3.2(-25) and Ca_V_3.2(+25)) were investigated in this study. Here we found no significant effect of kindling or Z944 treatment on the expression of Ca_V_3.1, total Ca_V_3.2, Ca_V_3.3 or of the Ca_V_3.2(-25) and Ca_V_3.2(+25) splice variants in the sham kindled, vehicle, ETX or Z944 treated groups in the hippocampus and amygdala (data not shown).

## Discussion

Here we demonstrate that blocking T-type Ca^2+^ channels with the novel drug, Z944, delayed seizure progression in the amygdala kindling model of TLE. Importantly, blocking T-type Ca^2+^ channels with Z944 was not effective at suppressing seizures in fully kindled rats, indicating that Z944 delays progression to kindling without an anti-seizure effect in this model. ETX, a lower-potency non-selective T-type Ca^2+^ channel blocker used clinically as an anti-seizure drug in patients with genetic generalized epilepsy, also demonstrated a trend towards delaying seizure severity in this model, but this was significantly less than that observed for Z944. ETX also showed no anti-seizure effects in the fully-kindled rats, consistent with its clinical profile of having no efficacy against seizures in patients with TLE. The results of this study indicate that pharmacologically inhibiting T-type Ca^2+^ channels with Z944 may be an effective disease modifying strategy to inhibit the development of TLE after an brain insult, or retard its progression, but is unlikely to have symptomatic anti-seizure effects in the established condition.

T-type Ca^2+^ channel expression have been shown to be increased in limbic structures in pilocarpine-induced SE [21] and T-type Ca^2+^ channel currents have been shown to be increased in the kindling and pilocarpine models of TLE [20, 21]. Further, multiple T-type Ca^2+^ channel splice variants have been described for each of the Ca_V_ T-type and are thought to be an important mechanism towards achieving temporal and spatial functional diversity required for normal cell functioning [10, 11, 13, 28]. There is increasing evidence that Ca^2+^ currents play an important role in the pathogenesis of acquired limbic epileptogenesis. It is believed that TLE progresses through three major phases of development; an acute phase wherein injury occurs, a latency period during which epileptogenesis is occurring, and a chronic epilepsy phase characterized by spontaneous recurrent seizures often with increasing frequency and drug resistance [29, 30]. During the latency period, intracellular Ca^2+^ levels remain elevated and initiates many second messenger effects that produce long-term plasticity changes in these neurons [10, 11, 13]. Paroxysmal depolarizing shifts and high frequency bursting of epileptiform activity has been associated with increased Ca^2+^ influx [31]. Furthermore, chronically epileptic neurons showed long-term alterations in Ca^2+^ homeostatic mechanisms [32] including higher Ca^2+^ levels and delayed recovery of resting Ca^2+^ levels when challenged with glutamate compared with controls in the pilocarpine-induced SE model of TLE [33].

A plausible mechanism for how Z944 delays the progression of seizure severity during kindling could be by inhibition of neuronal burst firing. T-type Ca^2+^ channels are critically important in generating low-threshold burst firing which is known to synchronize neuronal network activity and correlate with seizure activity in animal models of TLE [14, 15, 22]. In addition, blocking Ca^2+^ influx with Z944 may decrease the severity of the behavioural manifestation of the kindled seizure by reducing glutamate release. Furthermore, the blocking of excessive Ca^2+^ influx could also prevent changes in the γ-amino butyric acid (GABA) system, including decreased GABA_A_ receptor function [34], increased rate of GABA_A_ receptor endocytosis and decreased response to GABA [35] as has been shown in epileptic cultured hippocampal neurons. There are other effects of altered Ca^2+^ dynamics that could affect factors such as gene transcription, protein expression, neurogenesis, neuronal sprouting, as well as other physiological processes and secondary messenger/transduction pathways during epileptogenesis [36, 37]. In support of our results, several other studies have implicated alterations in Ca^2+^ channels and Ca^2+^ channel currents in the development of epilepsy in pilocarpine-induced SE [9, 14, 15, 21], as well as in amygdala kindled model of TLE [20].

Taken together, it is plausible that blocking of T-type Ca^2+^ channel function may lead to long-term changes in multiple neurobiological factors contributing to both excitability and gene expression, which may alter the excitability of the limbic system. This notion is supported by studies in the pilocarpine-induced SE model of TLE, wherein the SE promoted spontaneous firing of hippocampal CA1 neurons by selectively increasing the density of T-type Ca^2+^ currents [22]. In a subsequent study, focal application of a low concentration of nickel (to preferentially block Ca_V_3.2) or amiloride (a diuretic drug that inhibits T-type Ca^2+^ currents as well as having effects on Na^+^-H^+^ and Na^+^-Ca^2+^ exchangers) 7–21 days after pilocarpine-induced SE resulted in suppression of the SE-induced burst firing observed in CA1 hippocampal neuron apical dendrites [15]. Furthermore, a study by Becker and colleagues demonstrated that SE resulted in transient up-regulation of Ca_V_3.2 mRNA and protein expression in the CA1 region of the hippocampus. These changes subsequently caused an increase in T-type currents and burst-firing in wild-type mice but not in Ca_V_3.2 knock-out mice [21]. Importantly, Ca_V_3.2 knockout mice were resistant to the development of chronic seizures following pilocarpine-induced SE, and hippocampal sclerosis and mossy fibre sprouting, histopathological hallmarks of TLE in humans and animal models, were absent in these knock-out mice [21].

An increase in T-type Ca^2+^ currents has also been reported in the kindling model of TLE [20], however there are no reports in the literature documenting changes in the expression of T-type Ca^2+^ channels after amygdala kindling. Our hypothesis was that kindling would increase T-type Ca^2+^ expression and that treatment with Z944 would block this increase. We measured the mRNA expression of all three main T-type Ca^2+^ channels and two Ca_V_3.2 splice variants in the amygdala and hippocampus two to four weeks after the final kindling stimulation and found that neither kindling nor treatment with Z944 or ETX had any significant effect on the expression any of the T-type Ca^2+^ channels. T-type channel mRNA expression was examined two to four weeks after the end of kindling so as to rule out any acute effects of the seizures, however it is possible that changes in T-type Ca^2+^ channel expression may be transient, similar to that observed during the epileptogenesis phase in the pilocarpine-induced SE model [2121] and thus no longer observed at the longer time point assessed here. We did note a non-significant trend for increased mRNA expression of Ca_V_3.1 and Ca_V_3.3 in the amygdala and in all three T-type Ca^2+^ channels in the hippocampus in the kindled + vehicle group.

## Conclusion

The results of this study provide evidence that the selective, high potency, T-type Ca^2+^ channel blocker, Z944, is effective in delaying seizure progression in the amygdala kindling model. This may suggests that pharmacologically targeting T-type Ca^2+^ channels may be an effective disease modifying strategy to inhibit the onset or progression of TLE. However, further testing in other TLE models should be performed before the anti-epileptogenic role of Z944 is established.
